# Long-term exposure to ambient PM_2·5_, active commuting, and farming activity and cardiovascular disease risk in adults in China: a prospective cohort study

**DOI:** 10.1016/S2542-5196(23)00047-5

**Published:** 2023-04-03

**Authors:** Dong Sun, Cong Liu, Yinqi Ding, Canqing Yu, Yu Guo, Dianjianyi Sun, Yuanjie Pang, Pei Pei, Huaidong Du, Ling Yang, Yiping Chen, Xia Meng, Yang Liu, Jiben Liu, Rajani Sohoni, Gary Sansome, Junshi Chen, Zhengming Chen, Jun Lv, Haidong Kan, Liming Li

**Affiliations:** a Department of Epidemiology and Biostatistics, School of Public Health, Peking University, Beijing, China; b School of Public Health, Key Lab of Public Health Safety of the Ministry of Education, NHC 12 Key Lab of Health Technology Assessment, IRDR ICoE on Risk Interconnectivity and 13 Governance on Weather or Climate Extremes Impact and Public Health, Fudan University, Shanghai, China; c Peking University Center for Public Health and Epidemic Preparedness and Response, Beijing, China; d Fuwai Hospital Chinese Academy of Medical Sciences, Beijing, China; e Chinese Academy of Medical Sciences, Beijing, China; f Medical Research Council Population Health Research Unit, University of Oxford, Oxford, UK; g Clinical Trial Service Unit and Epidemiological Studies Unit, Nuffield Department of Population Health, University of Oxford, Oxford, UK; h Gangarosa Department of Environmental Health, Rollins School of Public Health, Emory University, Atlanta, GA, USA; i Prevention and Health Department, Yongqinglu Community Health Service, Qingdao, China; j China National Center for Food Safety Risk Assessment, Beijing, China

## Abstract

**Background:**

Increased physical activity is associated with a reduced risk of cardiovascular disease, but outdoor physical activity can be accompanied by increased inhalation of fine particulate matter (PM_2·5_). The extent to which long-term exposure to PM_2·5_ can offset the cardiovascular benefits of physical activity is unknown. We aimed to evaluate whether the associations between active commuting or farming activity and incident risks of cerebrovascular disease and ischaemic heart disease were consistent between populations with different ambient PM_2·5_ exposures.

**Methods:**

We did a prospective cohort study using data from people aged 30–79 years without cardiovascular disease at baseline from the China Kadoorie Biobank (CKB). Active commuting and farming activity were assessed at baseline using questionnaires. A high-resolution (1 × 1 km) satellite-based model was used to estimate annual average PM_2·5_ exposure during the study period. Participants were stratified according to PM_2·5_ exposure (54 μg/m^3^ or greater *vs* less than 54 μg/m^3^). Hazard ratios (HRs) and 95% CIs for incident cerebrovascular disease and ischaemic heart disease by active commuting and farming activity were estimated using Cox proportional hazard models. Effect modifications by PM_2·5_ exposure were tested by likelihood ratio tests. Analyses were restricted to the period from Jan 1, 2005, to Dec 31, 2017.

**Findings:**

Between June 25, 2004, and July 15, 2008, 512 725 people were enrolled in the CKB cohort. 322 399 eligible participants completed the baseline survey and were included in the analysis of active commuting (118 274 non-farmers and 204 125 farmers). Among 204 125 farmers, 2985 reported no farming time and 201 140 were included in the farming activity analysis. During a median follow-up of 11 years, 39 514 cerebrovascular disease cases and 22 313 ischaemic heart disease cases were newly identified. Among non-farmers with exposure to annual average PM_2·5_ concentrations of less than 54 μg/m^3^, increased active commuting was associated with lower risks of cerebrovascular disease (highest active commuting *vs* lowest active commuting HR 0·70, 95% CI 0·65–0·76) and ischaemic heart disease (0·60, 0·54–0·66). However, among non-farmers with exposure to annual average PM_2·5_ concentrations of 54 μg/m^3^ or greater, there was no association between active commuting and cerebrovascular disease or ischaemic heart disease. Among farmers with exposure to annual average PM_2·5_ concentrations of less than 54 μg/m^3^, increased active commuting (highest active commuting *vs* lowest active commuting HR 0·77, 95% CI 0·63–0·93) and increased farming activity (highest activity *vs* lowest activity HR 0·85, 95% CI 0·79–0·92) were both associated with a lower cerebrovascular disease risk. However, among farmers with exposure to annual average PM_2·5_ concentrations of 54 μg/m^3^ or greater, increases in active commuting (highest active commuting *vs* lowest active commuting HR 1·12, 95% CI 1·05–1·19) and farming activity (highest activity *vs* lowest activity HR 1·18, 95% CI 1·09–1·28) were associated with an elevated cerebrovascular disease risk. The above associations differed significantly between PM_2·5_ strata (all interaction p values <0·0001).

**Interpretation:**

For participants with long-term exposure to higher ambient PM_2·5_ concentrations, the cardiovascular benefits of active commuting and farming activity were significantly attenuated. Higher levels of active commuting and farming activity even increased the cerebrovascular disease risk among farmers with exposure to annual average PM_2·5_ concentrations of 54 μg/m^3^ or greater.

**Funding:**

National Natural Science Foundation of China, National Key Research and Development Program of China, Kadoorie Charitable Foundation, UK Wellcome Trust.

## Introduction

Being physically active is a well-established protective factor for cardiovascular health.^1^, ^2^ However, the inhaled dose of fine particulate matter (PM_2·5_) increases with ventilation frequency and exposure time during outdoor physical activity.^3^ Long-term PM_2·5_ exposure has been associated with an elevated risk of cardiovascular disease.^4^, ^5^ To what extent the beneficial effect of physical activity can be offset and even turn into increasing cardiovascular disease risk due to prolonged outdoor time and higher inhalation rates in settings with high PM_2·5_ concentrations remains a concern, with no clear conclusions.^6^

**Research in context**

**Evidence before this study**

We searched PubMed on Jan 15, 2023, for articles published from database inception to Jan 15, 2023, using the terms (“fine particulate matter” OR “PM_2·5_”) AND (“physical activity” OR “commuting” OR “farming”) AND (“cardiovascular” OR “cerebrovascular” OR “coronary” OR “myocardial infarction” OR “stroke”). We further searched Embase and Google Scholar using a similar search strategy. We did not apply restrictions on study type or language. Relevant studies were supplemented by reviewing the reference list of the searched articles. Previous studies on the effect modifications of physical activity on cardiovascular risk by long-term exposure to fine particulate matter (PM_2·5_) were mainly conducted in populations with relatively low PM_2·5_ exposure, with little variation in population characteristics (eg, living in an urban or rural area, and age), and included outdoor and indoor physical activity. The extent to which PM_2·5_ exposure in highly polluted regions can offset the cardiovascular benefits of active commuting and farming activity, mainly performed outdoors, remains inconclusive.

**Added value of this study**

We found that active commuting and farming activity were associated with reduced cardiovascular disease risk among participants with low PM_2·5_ exposure. More importantly, for the first time, we observed that the cardiovascular benefits of active commuting and farming activity were significantly attenuated with long-term exposure to high PM_2·5_ concentrations. Active commuting and farming activity were even associated with increased risk of cerebrovascular disease among farmers with high PM_2·5_ exposure. To our knowledge, this is by far the largest prospective study to comprehensively examine the effect modifications of active commuting and farming activity on cardiovascular disease risk by long-term exposure to PM_2·5_.

**Implications of all the available evidence**

The potential health impact of ambient air pollution has received more attention in urban areas than in rural areas. Our findings indicate that more stringent and sustained measures should be implemented to improve air quality in China, with similar efforts put into rural areas and urban areas. Future studies are warranted to explore the effect modifications by specific PM_2·5_ constitutions and other air pollutants.

Only a few studies have investigated the effect modifications of physical activity on cardiovascular disease risk by PM_2·5_ exposure,^7^, ^8^, ^9^, ^10^, ^11^ with most studies showing consistent associations across different strata by PM_2·5_ concentrations.^8^, ^9^, ^10^ However, these studies were mainly conducted in populations of high-income countries or regions, who were exposed to lower PM_2·5_ concentrations than most populations of low-income and middle-income countries.^8^, ^9^, ^10^, ^11^ Furthermore, most of these studies covered small geographical areas with little variation in population characteristics and did not differentiate between indoor and outdoor physical activity.

For the broader populations in low-income and middle-income countries, occupational and commuting activities contribute most to daily physical activity,^12^ including farming activity and active commuting (walking or cycling to work) outdoors. One previous study covering broad rural areas across China found no significant difference in the associations of commuting mode with cardiovascular disease incidence between groups with low PM_2·5_ exposure and high PM_2·5_ exposure.^7^ To our knowledge, no previous study has examined whether PM_2·5_ exposure can modify the association between outdoor farming activity and cardiovascular disease risk.

We aimed to evaluate whether the associations between active commuting or farming activity and incident risks of cerebrovascular disease and ischaemic heart disease were consistent between populations with different PM_2·5_ exposures.

## Methods

### Study design and participants

We did a prospective cohort study using data from the China Kadoorie Biobank (CKB). Details of the CKB have been published elsewhere.^13^ Briefly, the CKB included 0·5 million Chinese adults from urban and rural regions that are geographically spread across China and have large variations in PM_2·5_ concentrations. The baseline survey was conducted between June 25, 2004, and July 15, 2008. A total of 512 725 people aged 30–79 years were recruited from five urban and five rural areas across China, with 100–150 administrative units (rural villages or urban residential committees) included in each study area, and were invited to the local clinics within the administrative units to participate in the baseline survey. All the participants provided written informed consent and were asked to complete a laptop-based questionnaire, physical measurements, and blood draw. We excluded participants who had heart disease or stroke at baseline, developed cerebrovascular disease or ischaemic heart disease between baseline and Dec 31, 2004, had missing data for BMI or household air pollution, or reported implausible physical activity data. The Ethical Review Committee of the Chinese Center for Disease Control and Prevention (Beijing, China) and the Oxford Tropical Research Ethics Committee, University of Oxford (Oxford, UK) approved the study.

### Procedures

We assessed the occupational, commuting, domestic, and leisure-time physical activity at baseline by a questionnaire adapted from validated questionnaires used in previous studies^14^, ^15^ and further modified following a CKB pilot study. The questionnaire is shown in the appendix (pp 4–5), and has been described in a previous publication.^12^

We stratified the participants on the basis of occupation (farmer *vs* non-farmer), and asked different questions about commuting for non-farmers and farmers. Non-farmers with jobs reported one of the most frequently used commuting modes (single choice): walking; cycling; motorcycle or moped; car or bus, ferry, or train; or working at home or near home. Those who chose options other than working at home or near home further reported daily commute time (in minutes). For farmers, we only asked one question about the active commuting time, with no further distinction between cycling or walking. Consistent with previous studies,^16^ active commuting was defined as cycling or walking.

We asked farmers about their farming activity, starting by asking whether the farm work varied seasonally. For those with a seasonal work pattern, we asked about the duration of the busy seasons (in months), the main types of work (mainly manual, semi-mechanised, or mechanised), and daily farming time (in hours) in busy seasons, and weekly farming time (in hours) in quiet seasons. We asked about farming time (in hours) in a typical week for those whose farming activities were consistent across seasons. In addition to farming, we also asked farmers whether they had other off-farm jobs and the weekly working time (in hours).

The metabolic equivalent of task (MET) of a particular type of physical activity was assigned according to the 2011 compendium of physical activities (appendix pp 6–7).^17^, ^18^ Because we did not ask farmers about the commute time of cycling or walking separately, a mean MET value for cycling and walking was assigned to active commuting for farmers. The amount of each type of physical activity was derived by multiplying the corresponding MET value and hours spent per day and was further summed to get total physical activity (MET-h/day). Validations of the reproducibility of active commuting and farming activity are also shown in the appendix (p 8).

Other covariates included demographic characteristics, lifestyle factors, pollution from household fuel combustion, passive smoking, self-rated health status, physical measurements, personal medical history, and family medical histories. A detailed description of covariates is presented in the appendix (p 8).

We employed a high-resolution (1 × 1 km) satellite-based model to estimate daily PM_2·5_ concentrations for the study population.^19^ Briefly, this model incorporated the random-forest machine learning algorithms and linked the National Aeronautics and Space Administration multi-angle implementation of atmospheric correction aerosol optical depth (AOD) retrievals with daily PM_2·5_ measurements from more than 1300 ground monitors in China in 2013–17. Additional predictors were included according to previous studies in exposure assessment,^20^, ^21^ including meteorological parameters (temperature, relative humidity, wind speed, and pressure), land use variables (such as the normalised difference vegetation index), population density, and the modern-era retrospective analysis for research and applications PM_2·5_ products. An integrated model was built to enable gap-filling in regions with and without AOD values. The model performance indicated good consistency between predicted values and out-of-sample observations, with an overall cross-validation R^2^ of 0·87 (root-mean SE 16·1 μg/m^3^). The region-specific model performances were generally similar, with cross-validation R^2^ from 0·74 (root-mean SE 23·2 μg/m^3^) to 0·87 (11·0 μg/m^3^). Based on the established model, we predicted historical PM_2·5_ concentrations from 2005 to 2012. Annual PM_2·5_ concentrations were assigned to each participant based on the location of the clinic where the participant was recruited. Average annual PM_2·5_ concentrations from 2005 to the year of study outcome onset, death, loss to follow-up, or Dec 31, 2017 (whichever came first) were used as long-term PM_2·5_ exposure.

### Outcomes

Information on morbidity, hospitalisation, and mortality was collected through linking local disease and death registries and the national health insurance database. Causes of death were obtained from death certificates and supplemented by reviews of medical records and verbal autopsies. For participants who could not be linked to the health insurance database, we conducted annual follow-ups by review of residential records or visits to local communities. The follow-up of CKB participants is still ongoing. By the end of 2017, less than 1% of participants were lost to follow-up due to moving out of their baseline cities. Diseases and causes of death were coded using the International Classification of Diseases, 10th version (ICD-10). Outcomes of interest in this study were the incidence of fatal or non-fatal cerebrovascular disease (ICD-10 codes I60–I69) and ischaemic heart disease (I20–I25). A description of case adjudication is presented in the appendix (p 8).

### Statistical analysis

Analyses were restricted to the period from Jan 1, 2005, to Dec 31, 2017. Separate analyses were performed for non-farmers and farmers due to differences in commute types, occupational-related activities, and the corresponding baseline questions. For the analysis of active commuting, we excluded non-farmers who were retired or unemployed or worked at home, and farmers who reported neither farming time nor other off-farm jobs. For the analysis of farming activity, we further excluded farmers who reported no farming time.

Person-years at risk were calculated from the date of recruitment or Jan 1, 2005 (whichever came last) to the date of study outcome onset, death, loss to follow-up, or Dec 31, 2017 (whichever came first). Hazard ratios (HRs) and 95% CIs (CIs) were estimated using stratified Cox proportional hazard models, with age as the time scale and stratified by 5-year age groups and ten study areas to allow different baseline hazard functions for each stratum. Active commuting or farming activity was included in the models either using amount of activity or time. Restricted cubic splines with three knots were used to graphically estimate the associations of active commuting in both non-farmers and farmers and farming activity in farmers with study outcomes. Additionally, we divided the amount of physical activity (MET-h/day) from active commuting or farming activity into four groups, according to their distribution among non-farmers and farmers. A detailed description of model adjustment is presented in the appendix (pp 8–9). Proportional hazard assumptions were tested using Schoenfeld residuals, and no violation was detected.

The association analyses were performed in all eligible participants and in groups defined by dichotomous average annual PM_2·5_ concentration exposure. To ensure sufficient statistical power, we used the rounded median of PM_2·5_ exposure (54 μg/m^3^) as the cutoff, similar to three previous studies in China.^7^, ^9^, ^22^ Rather than using a single—somewhat arbitrary—cutoff, we also performed subgroup analyses with dichotomy by the upper tertile (56 μg/m^3^) of PM_2·5_ concentrations, as in previous studies.^10^, ^11^ Models with and without interaction terms of PM_2·5_ strata and physical activity were compared using likelihood ratio tests. Equations for these models are presented in the appendix (p 9).

Two-sided p values of less than 0·05 were considered significant. Restricted cubic splines were estimated using the R package rms and all figures were plotted in R (version 4.0.4). All other analyses were performed in Stata (version 15).

### Role of the funding source

The funders of the study had no role in study design, data collection, data analysis, data interpretation, writing of the report, or the decision to submit the manuscript for publication.

## Results

**Figure 1:**
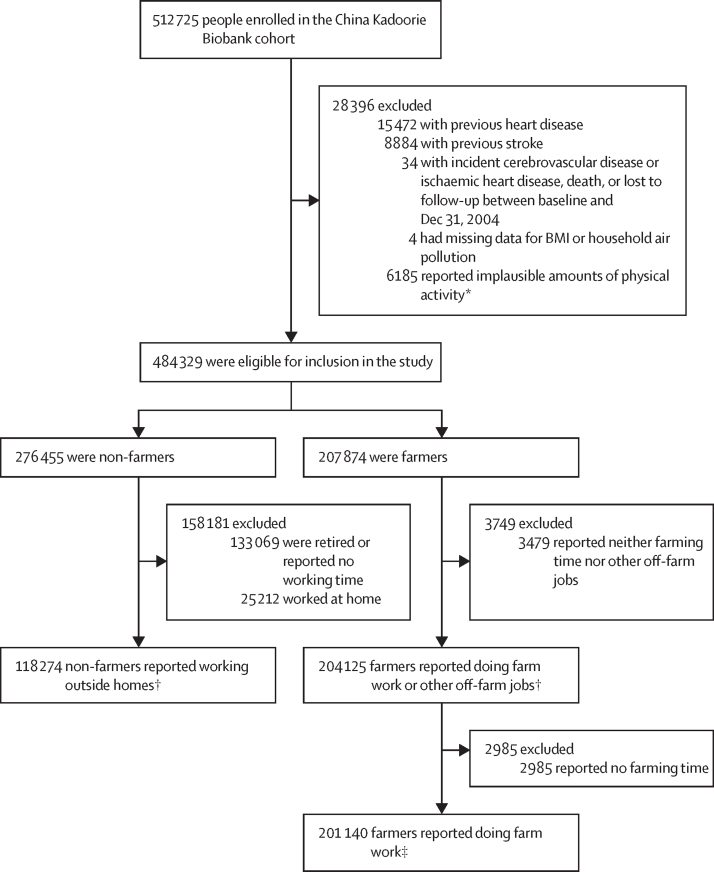
Study profile Numbers of participants excluded might not sum to the total number because some participants met more than one exclusion criterion. *Participants who did not report any physical activity, whose total time spent on physical activity and leisure sedentary activity was greater than 20 h, or who reported no work but commuting for work. †Included in the analyses of baseline characteristics and active commuting. ‡Included in the analysis of farming activity.

Between June 25, 2004, and July 15, 2008, 512 725 people were enrolled in the CKB cohort. 322 399 eligible participants completed the baseline survey and were included in the analysis of active commuting (118 274 non-farmers and 204 125 farmers; figure 1). Among 322 399 participants, 173 112 (53·7%) were female, 149 287 (46·3%) were male, and the mean age was 48·8 years (SD 9·4; table). Among 204 125 farmers, 2985 reported no farming time and 201 140 were included in the farming activity analysis.

**Table:** Baseline characteristics by the median of annual average PM_2·5_ concentration in the residential area

		**PM_2·5_<54 μg/m^3^(n=174 750)**	**PM_2·5_≥54 μg/m^3^ (n=147 649)**
Area of residence			
	Rural	141 588 (81·0%)	81 524 (55·2%)
	Urban	33 162 (19·0%)	66 125 (44·8%)
Sex			
	Female	96 521 (55·2%)	76 591 (51·9%)
	Male	78 229 (44·8%)	71 058 (48·1%)
Age, years		49·3 (9·7)	48·1 (9·0)
Highest level of education completed			
	Middle school or higher	70 805 (47·8%)	85 771 (49·5%)
Household income higher than 20 000 Chinese yuan		60 748 (39·1%)	70 460 (42·4%)
Married		161 812 (93·2%)	139 425 (93·8%)
Current smoker*		58 860 (34·0%)	50 810 (34·1%)
Current weekly alcohol consumption†		24 934 (16·7%)	31 768 (18·4%)
Daily food consumption‡			
	Red meat	39 342 (22·6%)	39 486 (26·7%)
	Fresh vegetables	157 450 (93·5%)	145 583 (96·0%)
	Fresh fruits	17 012 (12·9%)	28 258 (15·0%)
Physical activity, MET-h/day			
	Total physical activity	25·7 (17·6–34·9)	22·4 (14·2–35·4)
	Physical activity from active commuting§	1·8 (0·9–3·6)	0·0 (0·0–1·8)
	Physical activity from farming activity	11·7 (5·9–18·0)	3·2 (2·3–8·0)
	Leisure sedentary time, h/day	2·9 (1·4)	2·8 (1·5)
Cooking with solid fuels¶		81 987 (41·4%)	48 430 (39·1%)
Heating with solid fuels¶		78 820 (44·5%)	51 497 (37·1%)
Stove ventilation in baseline house		107 942 (56·5%)	70 640 (53·6%)
Self-rated good health		80 111 (49·5%)	79 818 (49·7%)
BMI, kg/m^2^		23·3 (3·1)	23·4 (3·3)
Waist–hip ratio		0·876 (0·068)	0·882 (0·071)
Hypertension		47 936 (28·1%)	47 655 (31·4%)
Diabetes		5293 (3·4%)	5660 (3·4%)
Family history of heart attack		4441 (2·7%)	5311 (3·4%)
Family history of stroke		23 983 (17·7%)	31 970 (17·1%)

Data are n (%), mean (SD), or median (IQR). All percentages and means were adjusted for age, sex, and study areas, except for these three variables. Differences between the two groups were tested by multiple linear regression for continuous variables and logistic regression for categorical variables, adjusted for age, sex, and study areas. All p values for differences between the two groups were less than 0·0001, except for current smokers (p=0·64), self-rated good health (p=0·39), diabetes (p=0·49), family history of stroke (p=0·020), and leisure sedentary time (p=0·0021). MET=metabolic equivalent of task.

* Including former smokers who had stopped smoking because of illness.

† Participants who reported weekly consumption of any volume of alcohol.

‡ Participants who reported daily consumption of any amount of each food.

§ Active commuting refers to cycling and walking.

¶ Solid fuels refer to wood and coal.

The average yearly PM_2·5_ concentrations during 2005–17 ranged from 26·2 μg/m^3^ (SD 0·4) in Haikou to 70·8 μg/m^3^ (1·4) in Henan (appendix pp 11–12). Participants with exposure to average annual PM_2·5_ concentrations of 54 μg/m^3^ or greater were more likely to live in urban areas and have lower total physical activity and physical activity from active commuting and farming than those with exposure to lower PM_2·5_ concentrations (table).

During a median follow-up of 11 years, 39 514 cases of incident cerebrovascular disease and 22 313 cases of incident ischaemic heart disease were identified.

**Figure 2:**
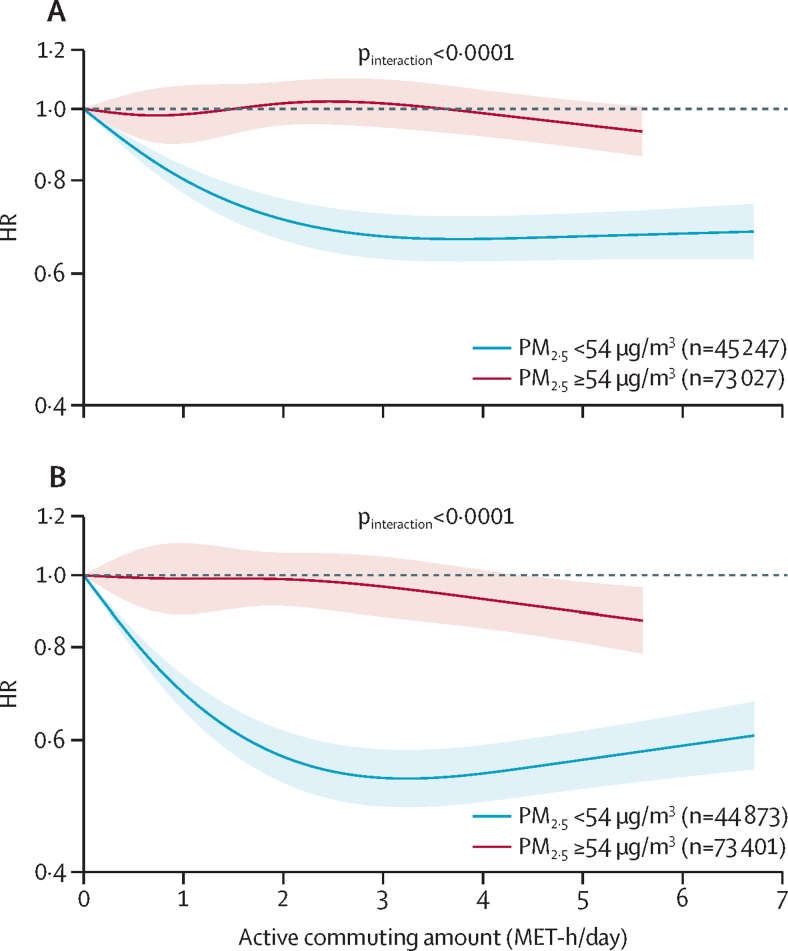
Associations of physical activity from active commuting with cerebrovascular disease and ischaemic heart disease among non-farmers stratified by the median of annual average PM_2·5_ concentration (n=118 274) (A) Cerebrovascular disease. (B) Ischaemic heart disease. Solid lines represent HRs, and the shaded areas represent 95% CIs. Curves within the 95th percentile of active commuting level in each stratum are shown. The models were adjusted for sex, age, education, household income, occupation, smoking status, alcohol consumption, consumption of fresh vegetables, fresh fruits, and red meat, leisure sedentary time, fuel types for cooking and heating in baseline house, cumulative exposure time to solid fuels in the past three houses, stove ventilation in the baseline house, duration of living with a smoker, exposure to second-hand smoke, BMI, waist–hip ratio, self-rated health status, hypertension and diabetes, family histories of heart disease and stroke, and the remaining activity amount after deducting active commuting from the total physical activity (MET-h/day). HR=hazard ratio. MET=metabolic equivalent of task.

Among non-farmers working outside homes, increased active commuting was associated with lower risks of cerebrovascular disease and ischaemic heart disease (appendix p 13). Among non-farmers with exposure to average annual PM_2·5_ concentrations of less than 54 μg/m^3^, increased active commuting was associated with lower risks of cerebrovascular disease (highest active commuting *vs* lowest active commuting HR 0·70, 95% CI 0·65–0·76) and ischaemic heart disease (0·60, 0·54–0·66), and the slopes flattened out with further increases in activity (figure 2; appendix p 14). By contrast, among non-farmers with exposure to average annual PM_2·5_ concentrations of 54 μg/m^3^ or greater, active commuting was not associated with cerebrovascular disease or ischaemic heart disease across the majority range of activity levels (figure 2; appendix p 14). The associations differed significantly between lower and higher PM_2·5_ exposure (both interaction p values <0·0001). Similar results were obtained by replacing amount of physical activity with active commute time (appendix p 15) or using the upper tertile of PM_2·5_ concentrations as the cutoff between strata (appendix p 16).

**Figure 3:**
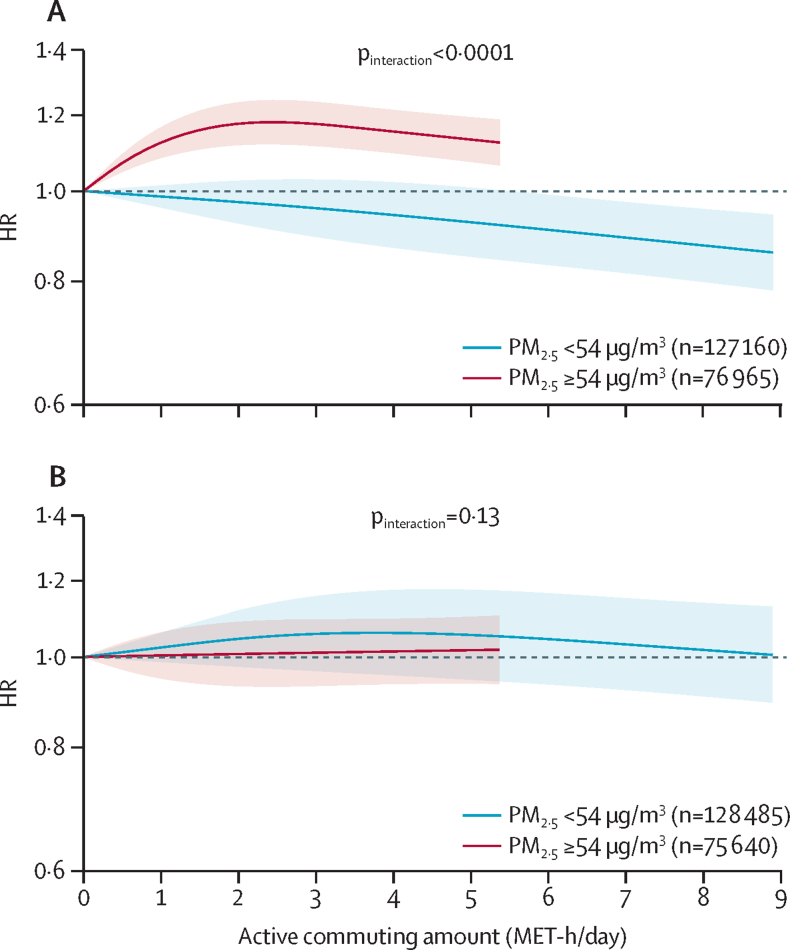
Associations of physical activity from active commuting with cerebrovascular disease and ischaemic heart disease among farmers stratified by the median of annual average PM_2·5_ concentration (n=204 125) (A) Cerebrovascular disease. (B) Ischaemic heart disease. Solid lines represent HRs, and the shaded areas represent 95% CIs. Curves within the 95th percentile of active commuting level in each stratum are shown. The models were adjusted for sex, age, education, household income, seasonal work pattern of farm, smoking status, alcohol consumption, consumption of fresh vegetables, fresh fruits, and red meat, leisure sedentary time, fuel types for cooking and heating in baseline house, cumulative exposure time to solid fuels in the past three houses, stove ventilation in the baseline house, duration of living with a smoker, exposure to second-hand smoke, BMI, waist–hip ratio, self-rated health status, hypertension and diabetes, family histories of heart disease and stroke, and the remaining activity amount after deducting active commuting from the total physical activity (MET-h/day). HR=hazard ratio. MET=metabolic equivalent of task.

Among farmers who reported farm work or other off-farm jobs, increased active commuting was associated with an increased risk of cerebrovascular disease but was not associated with the risk of ischaemic heart disease (appendix p 17). In the subgroup analyses according to the median of PM_2·5_ concentrations, there was a significant difference in the associations of active commuting with cerebrovascular disease risk between PM_2·5_ strata (interaction p<0·0001; figure 3; appendix p 18). Among farmers with exposure to average annual PM_2·5_ concentrations of less than 54 μg/m^3^, increased active commuting was associated with a reduced risk of cerebrovascular disease (highest active commuting *vs* lowest active commuting HR 0·77, 95% CI 0·63–0·93). However, among farmers with exposure to average annual PM_2·5_ concentrations of 54 μg/m^3^ or greater, increased active commuting was associated with an increased risk of cerebrovascular disease (compared with lowest active commuting; HRs from 1·22 [95% CI 1·15–1·30] to 1·12 [1·05–1·19]; appendix p 18). Among farmers, active commuting was not associated with ischaemic heart disease risk, regardless of PM_2·5_ concentrations. Similar results were obtained by replacing amount of physical activity with active commute time (appendix p 19) or using the upper tertile of PM_2·5_ as the cutoff between strata (appendix p 20).

**Figure 4:**
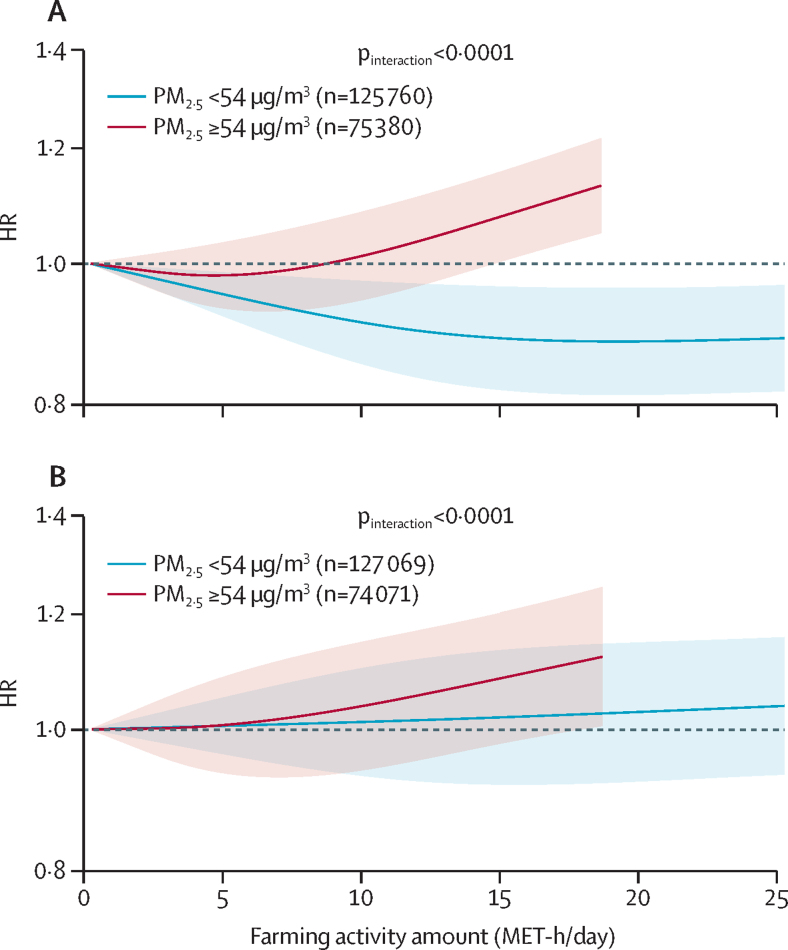
Associations of physical activity from farming with cerebrovascular disease and ischaemic heart disease among farmers stratified by the median of annual average PM_2·5_ concentration (n=201 140) (A) Cerebrovascular disease. (B) Ischaemic heart disease. Solid lines represent HRs, and the shaded areas represent 95% CIs. Curves within the 95th percentile of farming activity level in each stratum are shown. The models were adjusted for sex, age, education, household income, seasonal work pattern of farm, smoking status, alcohol consumption, consumption of fresh vegetables, fresh fruits, and red meat, leisure sedentary time, fuel types for cooking and heating in baseline house, cumulative exposure time to solid fuels in the past three houses, stove ventilation in the baseline house, duration of living with a smoker, exposure to second-hand smoke, BMI, waist–hip ratio, self-rated health status, hypertension and diabetes, family histories of heart disease and stroke, and the remaining activity amount after deducting farming activity from the total physical activity (MET-h/day). HR=hazard ratio. MET=metabolic equivalent of task.

Among farmers who reported farm work, increased farming activity was not associated with cerebrovascular disease or ischaemic heart disease risk (appendix p 21). In the analyses according to the median of annual average PM_2·5_ concentrations, increased farming activity was associated with a reduced risk of cerebrovascular disease among farmers with exposure to PM_2·5_ concentrations of less than 54 μg/m^3^ (highest activity *vs* lowest activity HR 0·85, 95% CI 0·79–0·92). However, risk of cerebrovascular disease increased with farming activity among farmers with exposure to PM_2·5_ concentrations of 54 μg/m^3^ or greater (highest activity *vs* lowest activity HR 1·18, 95% CI 1·09–1·28; interaction p<0·0001; figure 4; appendix p 22). Farming activity was not associated with ischaemic heart disease risk in each PM_2·5_ stratum. Similar results were obtained by replacing amount of physical activity with farming time (appendix p 23). The results were similar when using the upper tertile of PM_2·5_ as the cutoff between strata, except for a null association between farming activity and cerebrovascular disease among farmers exposed to PM_2·5_ concentrations of 56 μg/m^3^ or greater (appendix p 24).

## Discussion

In this large-scale prospective cohort study that included adults residing in areas with large variations in PM_2·5_ concentrations in China, we observed significant differences in associations of active commuting and farming activity with cardiovascular disease between participants with lower PM_2·5_ exposure and those with higher PM_2·5_ exposure. Among non-farmers with lower PM_2·5_ exposure, increased active commuting was associated with reduced risks of cerebrovascular disease and ischaemic heart disease. However, these associations disappeared for those with higher PM_2·5_ exposure. Among farmers with lower PM_2·5_ exposure, increased active commuting and farming activity were associated with a reduced risk of cerebrovascular disease. However, these activities were associated with an increased risk of cerebrovascular disease among farmers with higher PM_2·5_ exposure. Our findings suggest that higher PM_2·5_ exposure might counteract the protective cardiovascular effects of active commuting and farming activity.

Most studies on the trade-off between physical activity and PM_2·5_ exposure have not observed a significant difference in the association of physical activity with cardiovascular disease risk between lower PM_2·5_ exposure and higher PM_2·5_ exposure.^7^, ^8^, ^9^, ^10^ In the Nurses’ Health Study of 104 990 women (mean age 63 years), who were followed up for 20 years, no significant difference was observed in the associations of leisure-time physical activity with incident myocardial infarction and stroke by 24-month average PM_2·5_ exposure.^8^ Similar results were found in a cohort study of 58 643 older adults in Hong Kong with approximately 10 years of follow-up,^9^ and in a 3-year cohort analysis of 59 115 adults in South Korea.^10^ The possible reasons why these three previous studies did not observe effect modification by PM_2·5_ exposure include the relatively low concentrations and small variability in PM_2·5_, absence of a clear distinction between indoor and outdoor activities, and insufficient statistical power because of the small number of cases. In the China-PAR study of 76 176 adults who were widely distributed across rural China, the estimated effect of active commuting mode on reduced cardiovascular disease risk was larger among participants with lower PM_2·5_ exposure than those with higher exposure.^7^ However, no significant interaction of commuting mode (walking, cycling, or non-active commuting) and PM_2·5_ exposure was observed for cardiovascular disease incidence in this study (interaction p=0·055), which might be due to an insufficient number of cases.

A cohort study of 1 469 972 young adults in South Korea with a mean follow-up of 6 years found an association between changes in physical activity and cardiovascular disease risk that differed across exposure to lower PM_2·5_ concentrations and higher PM_2·5_ concentrations. Among participants with higher PM_2·5_ exposure, cardiovascular disease risk increased with an increase in physical activity above 1000 MET-min/week during two consecutive biennial health examinations compared with maintaining constant physical activity.^11^ Among participants with lower PM_2·5_ exposure, compared with maintaining constant physical activity, an increase in physical activity was not associated with cardiovascular disease risk. However, this study did not distinguish between indoor and outdoor physical activity and used baseline measurements from monitoring stations within the administrative residential districts as participants’ PM_2·5_ exposure, which might have led to relatively large misclassification bias.

In our study, the protective associations between active commuting and farming activity with cardiovascular disease found among participants with lower PM_2·5_ exposure were attenuated among those with higher PM_2·5_ exposure. The risk of cerebrovascular disease even increased with active commuting and farming activity among farmers with higher PM_2·5_ exposure. These findings are biologically reasonable. Engaging in physical activity improves antioxidant capacity, anti-inflammatory effects, and anti-atherosclerosis.^23^, ^24^ Additionally, physical activity improves cardiorespiratory fitness and is favourably associated with risk factors for cardiovascular disease, such as obesity, lipid profiles, and blood pressure.^24^ However, increased physical activity and extended outdoor time can lead to increased inhalation of PM_2·5_. Long-term exposure to PM_2·5_ induces oxidative stress and inflammation, which can further trigger insulin resistance, endothelial dysfunction, and the progression of atherosclerosis.^23^ Epidemiological studies have shown that long-term exposure to PM_2·5_ is associated with an elevated risk of cardiovascular disease.^4^, ^5^

The differences in results between farmers and non-farmers might partly be attributed to differences in the prevalence of other cardiovascular disease risk factors and the baseline cardiovascular disease risk level for people living in rural and urban areas in China,^25^ leading to different additional benefits (or risks) from higher physical activity. Additionally, the sources and chemical compositions of PM_2·5_ are different between urban and rural areas in China. In less urban regions of China, coal and biomass combustions contribute more to PM_2·5_ emissions.^26^, ^27^

Among farmers with low PM_2·5_ exposure, we did not observe an association of active commuting or farming activity with ischaemic heart disease risk. One possible reason is the presence of other local-specific pollutants from domestic, agricultural, industrial, and transport sources in these rural areas that increased ischaemic heart disease risk and related risk factors. Inhalation of these pollutants also increased with physical activity, which offset the favourable effect of physical activity on ischaemic heart disease. For example, previous meta-analyses have shown that long-term exposure to nitrogen oxides and ozone was associated with elevated ischaemic heart disease risk, but not stroke risk.^28^, ^29^ Additionally, two previous studies also did not observe an association between physical activity or changes in physical activity and coronary heart disease risk among participants with low PM_2·5_ exposure.^10^, ^11^

To our knowledge, this is the largest prospective study to comprehensively examine the effect modifications of active commuting and farming activity on cardiovascular disease risk by long-term exposure to PM_2·5_. The present study covered a wide range of geographical areas across China, with large variations in PM_2·5_ exposure. The large sample size and the number of cases provided sufficient statistical power to analyse subtypes of cardiovascular disease in non-farmers and farmers separately. The CKB survey collected comprehensive information on physical activity, including type, intensity, and duration. The collection of various other information, especially on smoking, exposure to second-hand smoke, and household air pollution, enabled control for potential confounders. The ways in which we ascertained study outcomes during more than 10 years of follow-up helped avoid information bias caused by self-reporting. The satellite-based model with high spatiotemporal resolutions allowed us to model historical PM_2·5_ exposures.

This study has several limitations. First, the yearly average concentrations of PM_2·5_ exposure were assigned according to the clinic locations near participants’ homes. There was also no information on PM_2·5_ along their commuting routes and around farm work locations. Exposure misclassification was inevitable. However, given the relatively small variability in PM_2·5_ concentrations within each study area and that PM_2·5_ concentrations were only used to stratify the population in this study, such differences in the exposures might not have had a substantial effect on the results. Second, commuting and farming activities were self-reported and collected once at baseline, and might have changed over time. However, the analysis strategy using updating physical activity information during follow-up might also suffer from reverse causality, as lifestyle habits might change after developing a disease.^30^ Third, we did not differentiate between walking and cycling among farmers, leading to a biased estimation of amount of physical activity. However, results from analyses of active commuting time were not materially changed, whether in non-farmers or farmers. Fourth, compared with urban areas, the scarcity of monitoring stations in rural areas made it more challenging to validate model predictions with real-time measurements. Expanding the monitoring network coverage in rural areas is essential to improve the model performance in future studies. Finally, considering that the participants in this study were exposed to relatively high PM_2·5_ concentrations, caution is needed in extrapolating our results to other study areas.

In conclusion, based on this large prospective cohort of adults in China, among participants with exposure to lower PM_2·5_ concentrations, active commuting was associated with a reduced risk of cardiovascular disease among non-farmers, and active commuting and farming activity were associated with a reduced risk of cerebrovascular disease among farmers. However, for participants with long-term exposure to higher PM_2·5_ concentrations, the cardiovascular benefits of active commuting were attenuated to the null among non-farmers. Both active commuting and farming activity were even associated with increased cerebrovascular disease risk among farmers with exposure to higher PM_2·5_ concentrations. Our findings have policy implications for continuously improving air quality, with similar efforts put into rural and urban areas, to maximise the cardiovascular protective effects of physical activity in China. Future studies are warranted, using low-cost and portable air pollution sensors, to improve personalised monitoring.

## Data sharing

Details of how to access CKB data and details of the data release schedule are available at https://www.ckbiobank.org/site/data+access.

## Declaration of interests

We declare no competing interests.
