# Supplementary material for: Long-term exposure to ambient PM2·5, active commuting, and farming activity and cardiovascular disease risk in adults in China: a prospective cohort study
Source: Lancet Planet Health. 2023 Apr 3;7(4):e304–12. doi: 10.1016/S2542-5196(23)00047-5 (PMC10104773; doi:10.1016/S2542-5196(23)00047-5)
Supplement: Supplementary appendix [file mmc1.pdf]

THE LANCET
Planetary Health

Supplementary appendix

This appendix formed part of the original submission and has been peer reviewed. We post it as supplied by the authors.

Supplement to: Sun D, Liu C, Ding Y, et al. Long-term exposure to ambient $PM_{2·5}$, active commuting, and farming activity and cardiovascular disease risk in adults in China: a prospective cohort study. *Lancet Planet Health* 2023; **7:** e304–12.

Appendix

Members of the China Kadoorie Biobank collaborative group ... 3

eMethods ... 4

Questionnaire on physical activity used in the CKB study at baseline ... 4
Link to the electronic questionnaires ... 5
Physical activity types, MET values, codes and intensity categories ... 6
Reproducibility of active commuting and farming activity ... 8
Description of covariates ... 8
Case Adjudication ... 8
Model adjustment ... 8
Model equation ... 9
Reference ... 10

Table S1. Average yearly $PM_{2.5}$ concentrations (μg/m^3) from 2005 to 2017 among ten study areas ... 11

Figure S1. Geographical locations of 10 study areas and estimated average $PM_{2.5}$ concentrations in China from 2005 to 2017 ... 12

Figure S2. Associations of physical activity from active commuting with cerebrovascular disease and ischemic heart disease among non-farmers (n=118,274). ... 13

Figure S3. Associations of active commuting level categories with cerebrovascular disease and ischemic heart disease among non-farmers stratified according to the median of $PM_{2.5}$ concentration (n=118,274). ... 14

Figure S4. Associations of active commute time with cerebrovascular disease and ischemic heart disease among non-farmers stratified according to the median of $PM_{2.5}$ concentration (n=118,274). ... 15

Figure S5. Associations of physical activity from active commuting with cerebrovascular disease and ischemic heart disease among non-farmers stratified according to the upper tertile of $PM_{2.5}$ concentration (n=118,274). ... 16

Figure S6. Associations of physical activity from active commuting with cerebrovascular disease and ischemic heart disease among farmers (n=204,125). ... 17

Figure S7. Associations of active commuting level categories with cerebrovascular disease and ischemic heart disease among farmers stratified according to the median of $PM_{2.5}$ concentration (n=204,125). ... 18

Figure S8. Associations of active commute time with cerebrovascular disease and ischemic heart disease among farmers stratified according to the median of $PM_{2.5}$ concentration (n=204,125). ... 19

Figure S9. Associations of physical activity from active commuting with cerebrovascular disease and ischemic heart disease among farmers stratified according to the upper tertile of $PM_{2.5}$ concentration (n=204,125). ... 20

Figure S10. Associations of physical activity from farming with cerebrovascular disease and ischemic heart disease among farmers (n=201,140). 21

Figure S11. Associations of farming activity level categories with cerebrovascular disease and ischemic heart disease among farmers stratified according to the median of $PM_{2.5}$ concentration (n=201,140). 22

Figure S12. Associations of farming time with cerebrovascular disease and ischemic heart disease among farmers stratified according to the median of $PM_{2.5}$ concentration (n=201,140). 23

Figure S13. Associations of physical activity from farming with cerebrovascular disease and ischemic heart disease among farmers stratified according to the upper tertile of $PM_{2.5}$ concentration (n=201,140). 24

Members of the China Kadoorie Biobank collaborative group

International Steering Committee: Junshi Chen, Zhengming Chen (PI), Robert Clarke, Rory Collins, Yu Guo, Liming Li (PI), Jun Lv, Richard Peto, Robin Walters. **International Co-ordinating Centre, Oxford:** Daniel Avery, Derrick Bennett, Ruth Boxall, Sue Burgess, Ka Hung Chan, Yumei Chang, Yiping Chen, Zhengming Chen, Johnathan Clarke; Robert Clarke, Huaidong Du, Ahmed Edris Mohamed, Zammy Fairhurst-Hunter, Hannah Fry, Simon Gilbert, Alex Hacker, Mike Hill, Michael Holmes, Pek Kei Im, Andri Iona, Maria Kakkoura, Christiana Kartsonaki, Rene Kerosi, Kuang Lin, Mohsen Mazidi, Iona Millwood, Sam Morris, Qunhua Nie, Alfred Pozarickij, Paul Ryder, Saredo Said, Sam Sansome, Dan Schmidt, Paul Sherliker, Rajani Sohoni, Becky Stevens, Iain Turnbull, Robin Walters, Lin Wang, Neil Wright, Ling Yang, Xiaoming Yang, Pang Yao.

National Co-ordinating Centre, Beijing: Yu Guo, Xiao Han, Can Hou, Jun Lv, Pei Pei, Chao Liu, Canqing Yu, Qingmei Xia. **10 Regional Co-ordinating Centres: Qingdao CDC:** Zengchang Pang, Ruqin Gao, Shanpeng Li, Haiping Duan, Shaojie Wang, Yongmei Liu, Ranran Du, Yajing Zang, Liang Cheng, Xiaocao Tian, Hua Zhang, Yaoming Zhai, Feng Ning, Xiaohui Sun, Feifei Li. **Licang CDC:** Silu Lv, Junzheng Wang, Wei Hou. **Heilongjiang Provincial CDC:** Wei Sun, Shichun Yan, Xiaoming Cui. **Nangang CDC:** Chi Wang, Zhenyuan Wu,Yanjie Li, Quan Kang. **Hainan Provincial CDC:** Huiming Luo, Tingting Ou. **Meilan CDC:** Xiangyang Zheng, Zhendong Guo, Shukuan Wu, Yilei Li, Huimei Li. **Jiangsu Provincial CDC:** Ming Wu, Yonglin Zhou, Jinyi Zhou, Ran Tao, Jie Yang, Jian Su. **Suzhou CDC:** Fang Liu, Jun Zhang, Yihe Hu, Yan Lu, Liangcai Ma, Aiyu Tang, Shuo Zhang, Jianrong Jin, Jingchao Liu. **Guangxi Provincial CDC:** Mei Lin, Zhenzhen Lu. **Liuzhou CDC:** Lifang Zhou, Changping Xie, Jian Lan,Tingping Zhu,Yun Liu, Liuping Wei, Liyuan Zhou, Ningyu Chen, Yulu Qin, Sisi Wang. **Sichuan Provincial CDC:** Xianping Wu, Ningmei Zhang, Xiaofang Chen, Xiaoyu Chang. **Pengzhou CDC:** Mingqiang Yuan, Xia Wu, Xiaofang Chen, Wei Jiang, Jiaqiu Liu, Qiang Sun. **Gansu Provincial CDC:** Faqing Chen, Xiaolan Ren, Caixia Dong. **Maiji CDC:** Hui Zhang, Enke Mao, Xiaoping Wang, Tao Wang, Xi zhang. **Henan Provincial CDC:** Kai Kang, Shixian Feng, Huizi Tian, Lei Fan. **Huixian CDC:** XiaoLin Li, Huarong Sun, Pan He, Xukui Zhang. **Zhejiang Provincial CDC:** Min Yu, Ruying Hu, Hao Wang. **Tongxiang CDC**: Xiaoyi Zhang, Yuan Cao, Kaixu Xie, Lingli Chen, Dun Shen. **Hunan Provincial CDC:** Xiaojun Li, Donghui Jin, Li Yin, Huilin Liu, Zhongxi Fu. **Liuyang CDC:** Xin Xu, Hao Zhang, Jianwei Chen,Yuan Peng, Libo Zhang, Chan Qu.

eMethods

Questionnaire on physical activity used in the CKB study at baseline

- **Section A: For non-farmers**

1. In the past 12 months, how active were you at work?

☐ Mainly sedentary (e.g. office worker)
☐ Standing occupation (e.g. guard, shop assistant)
☐ Manual work (e.g. plumber, carpenter)
☐ Heavy manual work (e.g. miner, construction worker)
☐ Retired, housewife/husband, unemployed, or disabled → ***go to Question 12***

2. In a typical week, about how many hours did you usually work? ___hours

3. In the past 12 months, how did you usually get to work?

☐ Mainly walk ☐ By bicycle
☐ By motorbike ☐ By bus/car/ferry/train
☐ Mainly stayed at home, or work near home → ***go to Question 12***

4. How much time did you spend each day on the journey to and from work? ___mins

- **Section B: For farmers**

5. In the past 12 months, did your farming work change seasonally?

☐ No → ***go to Question 7***
☐ Yes

6. During the farming season in the last 12 months:

— How many months did it usually last? ___months
— What types of work did it usually involve?
☐ Manual ☐ Semi-mechanized ☐ Fully mechanized
— How many hours did you usually work each day? ___hours
— Of which, how many hours did you sweat or have a much faster heartbeat?
___hours

7. In a typical week, how many hours did you usually work in the field? ___hours

8. Apart from the agriculture work, did you have any other job?

☐ No → ***go to Question 11***
☐ Yes

9. How active were you at work with other job?

☐ Mainly sedentary ☐ Mainly general manual work
☐ Mainly standing ☐ Mainly heavy manual work

10. In a typical week, about how many hours did you work at other job? ___hours

11. In a typical day how much time did you usually spend on the journey to and from work on foot or by bicycle? ___mins

- **Section C: For both farmers and non-farmers**

12. During the past 12 months, how often did you exercise in your leisure time?

☐ Never or almost never
☐ 1-3 times/month
} → ***go to Question 15***

☐ 1-2 times/week
☐ 3-5 times/week
☐ Daily or almost every day

13. What is your main type of exercise? ***(tick one box only)***

☐ Taichi/Qigong ☐ Walking
☐ Jogging/aerobic exercise ☐ Swimming
☐ Ball games (basketball, table tennis, etc.) ☐ Other (eg. mountain climbing)

14. About how many hours per week did you spent on these exercises? ____hours

15. In a typical week during the past 12 months, how often did you sweat or have a much faster heartbeat because of physical activities/exercise?

☐ Never or almost never
☐ <1 time/week
} → ***go to Question 17***
☐ 1-2 times/week
☐ 3-5 times/week
☐ Daily or almost every day

16. About how many hours per week did you do such vigorous activities? ____hours

17. About how many hours per week did you do housework? ____ hour

Link to the electronic questionnaires

https://www.ckbiobank.org/site/binaries/content/assets/resources/pdf/qs_baseline-final-from10june2004.pdf

Physical activity types, MET values, codes and intensity categories

Activity type	Intensity	MET	Codes[*]
Heavy manual work	Vigorous	6.5	11477
Manual work	Moderate	4.5	11476
Standing work	Moderate	3.8	Mean of 11610 and 11630
Sedentary work	Low	1.8	Mean of 11580, 11585, and 11590
Manual work in the farming season	Vigorous	6.3	Mean of 11145 and 11146
Semi-mechanized work in the farming season	Moderate	3.4	Mean of 11146 and 11147
Fully mechanized work in the farming season	Low	2.4	Mean of 11147 and 11170
Work outside the farming season	Low	2.0	11147
Walking	Moderate	4.0	17270
Bicycle	Vigorous	6.8	1011
Motorbike	Moderate	3.5	16030
Private or public transportation (such as bus, car, underground, and ferry)	Low	1.7	Mean of 16010, 16015, and 16016
Household activity	Low	2.8	Mean of 05030[†], 05040[†], 05035, 05055, 05070, 05090[†], 05092[†], 05184, 05197, and 05200
Tai-Chi/qigong/leisure walking	Moderate	3.3	Mean of 15670 and 17160
Jogging/aerobic exercise	Vigorous	7.4	Mean of 03015, 12020, and 12150
Ball games	Moderate	5.5	Mean of 15020[†], 15030[†], 15055, 15080, 15090, 15255, 15605[†], 15610[†], 15652, 15660, 15675, 15710[†], and 15711[†]
Brisk walking/gymnastics/folk dancing	Moderate	4.2	Mean of 03025, 15300, and 17200
Swimming	Vigorous	7.2	Mean of 18230, 18240, and 18310
Other exercise, e.g. mountain walking, home exercise and rope jumping	Moderate	5.9	Mean of 02010, 02064, 04001, 04100, 15110[†], 15120[†], 15200, 15240, 15310, 15425[†], 15430[†], 15537, 15550[‡], 15551[‡], 15552[‡], 15580, 15590, 15730, 15732[‡], 15733[‡], 15734[‡], and 19030

MET, metabolic equivalent of task.

[*]Based on the 2011 Compendium of Physical Activities: a second update of codes and MET values. Ainsworth BE, et al. Medicine and Science in Sports and Exercise, 2011;43(8):1575-1581.

[†]Assigned 1/2 weight in calculating the mean MET value because the connecting two items represent one type of activity.

‡Assigned 1/3 weight in calculating the mean MET value because the connecting three items represent one type of activity.

Reproducibility of active commuting and farming activity

After the baseline survey, about 5% of the participants were randomly selected for the first resurvey in 2008. Reproducibility of the physical activity levels from active commuting and farming activity was tested in 3,402 participants who worked outside home at baseline and completed the same questionnaire within two years (median interval=1.7 years). The intraclass correlation coefficients for physical activity levels from active commuting and farming activity were 0.54 and 0.75, respectively.

Description of covariates

Other covariates collected using the baseline questionnaire included demographic characteristics (sex, age, educational level, income, and occupation), lifestyle factors (smoking and alcohol drinking status, consumption of red meat, fresh vegetables and fruits, and leisure sedentary time), pollution from household fuel combustion (solid fuel use for cooking and heating in the past three houses, and cookstove ventilation in the baseline house), passive smoking, self-rated health status, personal medical history, and family histories of diseases.

Physical measurements, including height, weight, waist and hip circumferences, and blood pressure, were conducted by trained staff using calibrated instruments. Body mass index (BMI) was calculated as weight (kilogram) divided by height (meter) squared. Waist-hip ratio (WHR) was calculated as waist circumference divided by hip circumference. Prevalent hypertension was defined as having measured systolic blood pressure≥140mmHg, diastolic blood pressure≥90mmHg, self-reported diagnosis of hypertension, or taking antihypertensive drugs. Prevalent diabetes was defined as having random blood glucose≥11.1mmol/L, fasting glucose≥7.0mmHg, self-reported diagnosis of diabetes, or taking anti-diabetic drugs. A family history of heart attack or stroke was defined as at least one of the parents and siblings with the corresponding disease.

Case Adjudication

As of 2018, medical records of 33,515 IHD cases and 38,823 stroke cases were retrieved and reviewed by cardiovascular specialists. The diagnosis was confirmed for 87.9% of IHD cases and 91.1% of stroke cases. [1]

Model adjustment

The model was adjusted for sex, age (years), education (no formal school, primary school, middle school, or high school or above), household income (<10000, 10000-19999, or ≥20000 RMB/y), occupation (manufacturing workers, managers, professionals, sales or service workers, self-employed, or others; only adjusted in analyses for non-farmers), seasonal work pattern of farm (have or not have, only adjusted in farmers), smoking status (non-smokers, former smokers who had stopped for reasons other than illness, current smokers or former smokers who had stopped because of illness: 1-14, 15-24, or ≥25 cigarettes or equivalent per day), alcohol consumption (less than weekly, former weekly, weekly but not daily, daily drinking <30, 30-59, or ≥60 g/day of pure alcohol), consumption of fresh vegetables, fresh fruits, and red meat (days per week), leisure sedentary time (hours per day), fuel types for cooking and heating in baseline house (solid fuels, clean fuels, other fuels, or heating/cooking less than once a month), cumulative exposure time to solid fuels in the past three houses (years), cookstove ventilation in the baseline house (have or not have), duration of living with a smoker (years), exposure

to secondhand smoke (hours/day), BMI (kg/m^2), WHR, self-rated health status (excellent, good, fair, or poor), prevalent hypertension and diabetes (yes or no), family histories of heart disease and stroke (have or not have), and the remaining part after deducting the active commuting or farming activity from the total physical activity (MET-h/d).

Model equation

We estimated the associations between physical activity and CVD using Cox proportional hazard models, stratified by the 5-year age groups and 10 study areas. For each participant, the hazard under this model is

$$\boldsymbol{h(t|x) = h_{0S}(t)exp(X_1\beta_1 + C\,\beta_C)}$$

where $\boldsymbol{h_{0S}(t)}$ is the baseline hazard for the stratum $\boldsymbol{S}$ defined by the 5-year age groups and 10 study areas, $\boldsymbol{X_1}$ is active commuting or farming activity, $\boldsymbol{C}$ are all the covariates, and $\boldsymbol{\beta_1}$ and $\boldsymbol{\beta_C}$ are coefficients for them.

In the subgroup analyses, we split the study population (farmers or non-farmers) into two subgroups by the median (or upper tertile in the sensitivity analysis) of $PM_{2.5}$ exposure. We performed the above stratified Cox models in each subgroup. We used likelihood ratio tests to compare the models with and without the interaction terms of the $PM_{2.5}$ strata and physical activity. The models with and without the interaction term are

$$\boldsymbol{h(t|x) = h_{0S}(t)exp(X_1\,\beta_1 + X_2\,\beta_2 + X_1\,X_2\,\beta_3 + C\,\beta_C)}$$

$$\boldsymbol{h(t|x) = h_{0S}(t)exp(X_1\,\beta_1 + X_2\,\beta_2 + C\,\beta_C)}$$

where $\boldsymbol{X_2}$ is the $PM_{2.5}$ strata, and $\boldsymbol{X_1\,X_2}$ is the interaction term of the $PM_{2.5}$ strata and active commuting or farming activity.

Reference

1. Turnbull I, Clarke R, Wright N, et al. Diagnostic accuracy of major stroke types in Chinese adults: A clinical adjudication study involving 40,000 stroke cases. *Lancet Reg Health West Pac* 2022; **21**: 100415.

Table S1. Average yearly $PM_{2.5}$ concentrations ($\mu g/m^3$) from 2005 to 2017 among ten study areas

	**Urban areas**					**Rural areas**				
	Qingdao	Harbin	Haikou	Suzhou	Liuzhou	Sichuan	Gansu	Henan	Zhejiang	Hunan
Mean±SD	57.1±1.0	55.3±1.3	26.2±0.4	57.4±1.7	45.3±0.9	51.4±4.0	39.0±2.0	70.8±1.4	53.5±1.1	51.2±1.7
P25	56.2	54.3	26.0	56.1	45.0	48.8	37.8	70.0	52.3	50.2
P50	57.2	55.5	26.3	58.0	45.4	51.9	39.1	70.7	53.5	51.2
P75	57.8	56.6	26.4	58.7	45.8	54.3	40.2	71.4	54.4	52.9

SD, standard derivation; P25, 25th percentile; P50, 50th percentile; P75, 75th percentile.

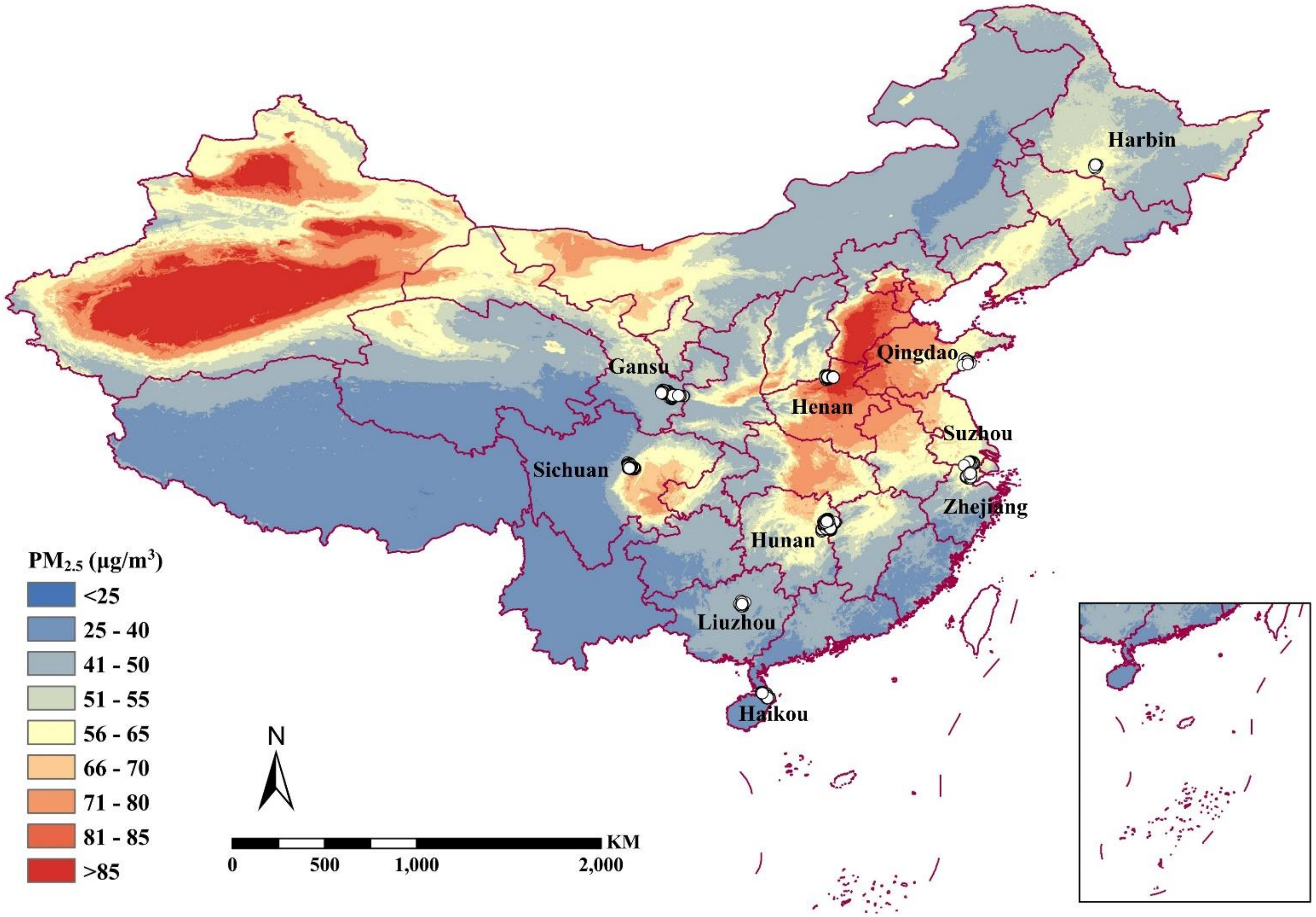

Figure S1. Geographical locations of 10 study areas and estimated average $PM_{2.5}$ concentrations in China from 2005 to 2017

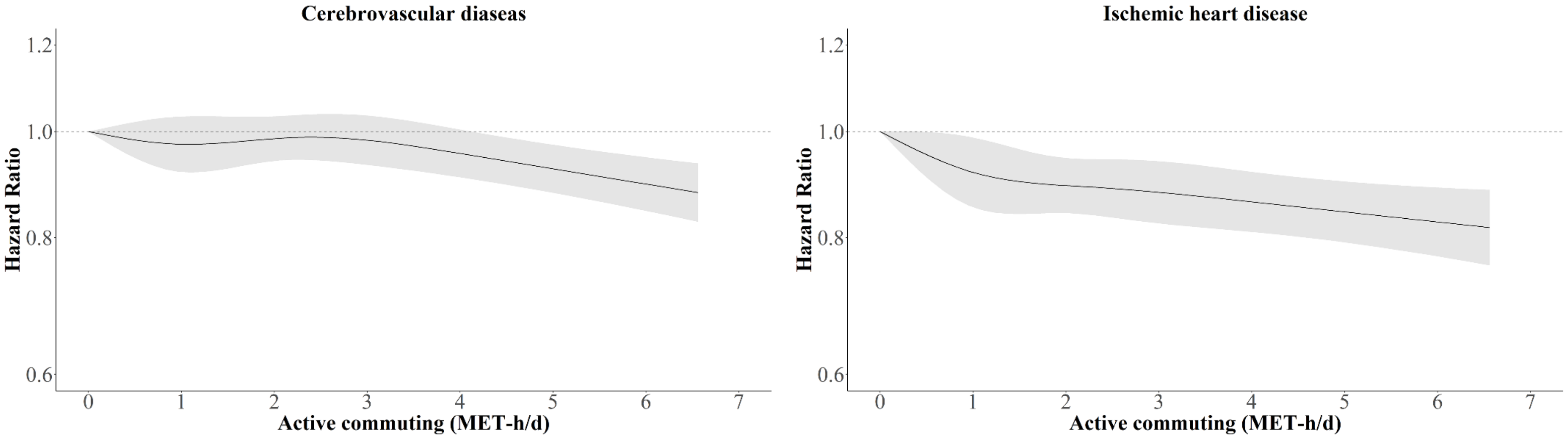

Figure S2. Associations of physical activity from active commuting with cerebrovascular disease and ischemic heart disease among non-farmers (n=118,274).
MET, metabolic equivalent of task. Solid lines represent hazard ratios and the ribbons represent 95% confidence intervals from Cox proportional hazard models with restricted cubic splines. Curves within the 95th percentile of active commuting level were shown.

The models were adjusted for sex, age (years), education, household income, occupation, smoking status, alcohol consumption, consumption of fresh vegetables, fresh fruits, and red meat (days per week), leisure sedentary time (hours per day), fuel types for cooking and heating in baseline house, cumulative exposure time to solid fuels in the past three houses (years), cookstove ventilation in the baseline house, duration of living with a smoker (years), exposure to secondhand smoke (hours/day), BMI (kg/m^2), WHR, self-rated health status, prevalent hypertension and diabetes, family histories of heart disease and stroke, and the remaining part after deducting the active commuting from the total physical activity (MET-h/d).

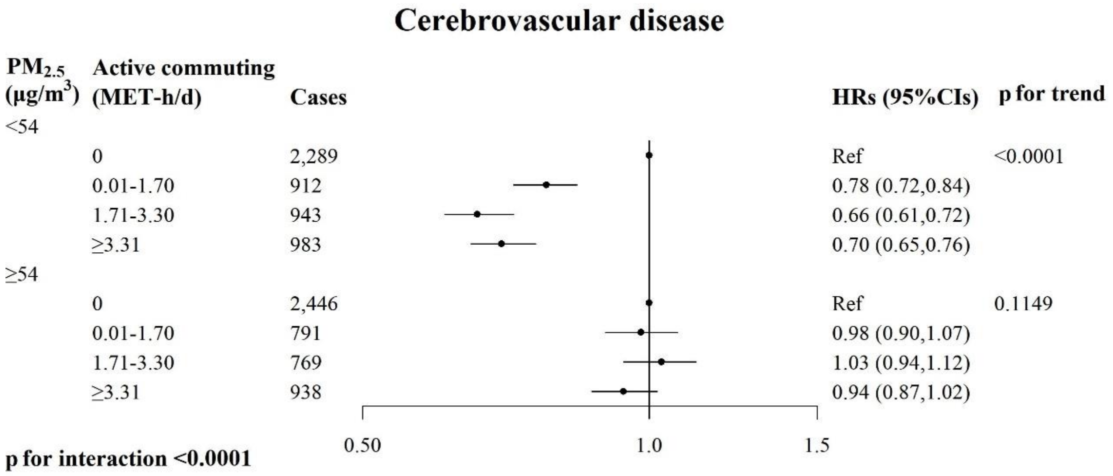

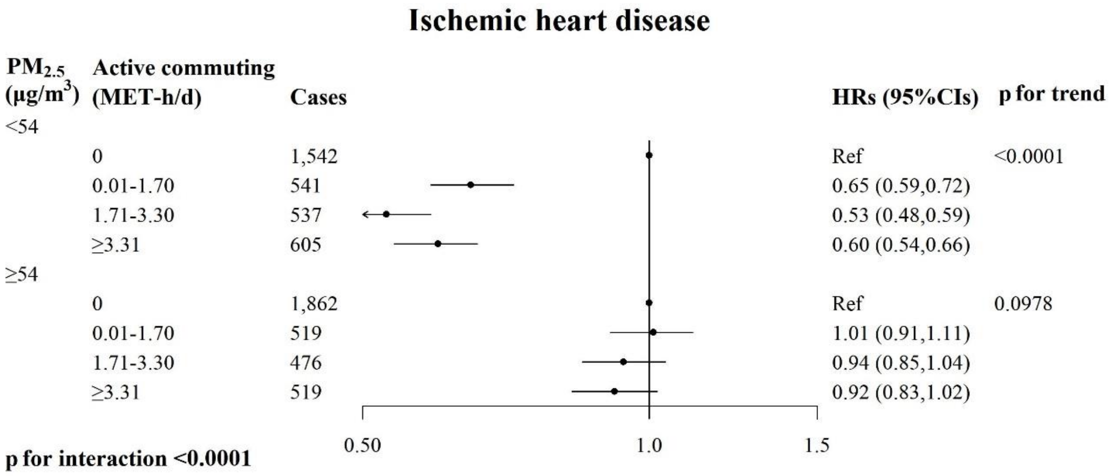

Figure S3. Associations of active commuting level categories with cerebrovascular disease and ischemic heart disease among non-farmers stratified according to the median of $PM_{2.5}$ concentration (n=118,274).

MET, metabolic equivalent of task.

The models were adjusted for sex, age (years), education, household income, occupation, smoking status, alcohol consumption, consumption of fresh vegetables, fresh fruits, and red meat (days per week), leisure sedentary time (hours per day), fuel types for cooking and heating in baseline house, cumulative exposure time to solid fuels in the past three houses (years), cookstove ventilation in the baseline house, duration of living with a smoker (years), exposure to secondhand smoke (hours/day), BMI (kg/m^2), WHR, self-rated health status, prevalent hypertension and diabetes, family histories of heart disease and stroke, and the remaining part after deducting the active commuting from the total physical activity (MET-h/d).

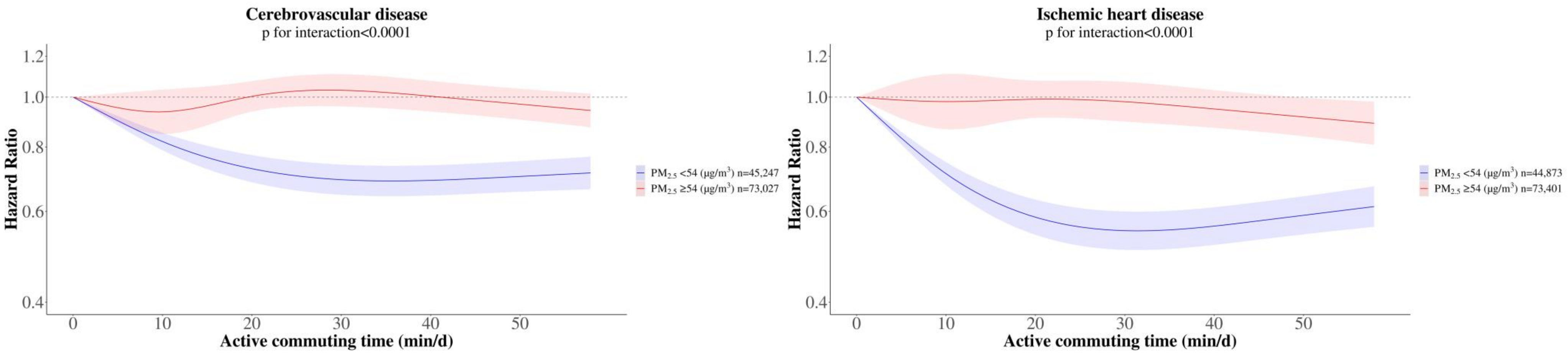

Figure S4. Associations of active commute time with cerebrovascular disease and ischemic heart disease among non-farmers stratified according to the median of $PM_{2.5}$ concentration (n=118,274).

MET, metabolic equivalent of task. Solid lines represent hazard ratios and the ribbons represent 95% confidence intervals from Cox proportional hazard models with restricted cubic splines. Curves within the 95th percentile of active commuting time in each stratum were shown.

The models were adjusted for sex, age (years), education, household income, occupation, smoking status, alcohol consumption, consumption of fresh vegetables, fresh fruits, and red meat (days per week), leisure sedentary time (hours per day), fuel types for cooking and heating in baseline house, cumulative exposure time to solid fuels in the past three houses (years), cookstove ventilation in the baseline house, duration of living with a smoker (years), exposure to secondhand smoke (hours/day), BMI (kg/m^2), WHR, self-rated health status, prevalent hypertension and diabetes, family histories of heart disease and stroke, and the remaining part after deducting the active commuting from the total physical activity (MET-h/d).

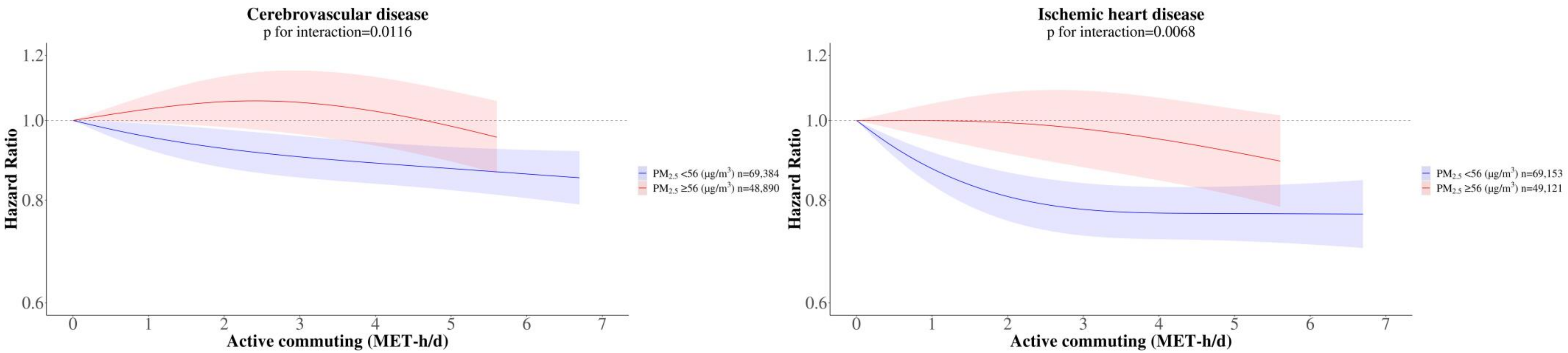

Figure S5. Associations of physical activity from active commuting with cerebrovascular disease and ischemic heart disease among non-farmers stratified according to the upper tertile of $PM_{2.5}$ concentration (n=118,274).

MET, metabolic equivalent of task. Solid lines represent hazard ratios and the ribbons represent 95% confidence intervals from Cox proportional hazard models with restricted cubic splines. Curves within the 95th percentile of active commuting level in each stratum were shown.

The models were adjusted for sex, age (years), education, household income, occupation, smoking status, alcohol consumption, consumption of fresh vegetables, fresh fruits, and red meat (days per week), leisure sedentary time (hours per day), fuel types for cooking and heating in baseline house, cumulative exposure time to solid fuels in the past three houses (years), cookstove ventilation in the baseline house, duration of living with a smoker (years), exposure to secondhand smoke (hours/day), BMI (kg/m^2), WHR, self-rated health status, prevalent hypertension and diabetes, family histories of heart disease and stroke, and the remaining part after deducting the active commuting from the total physical activity (MET-h/d).

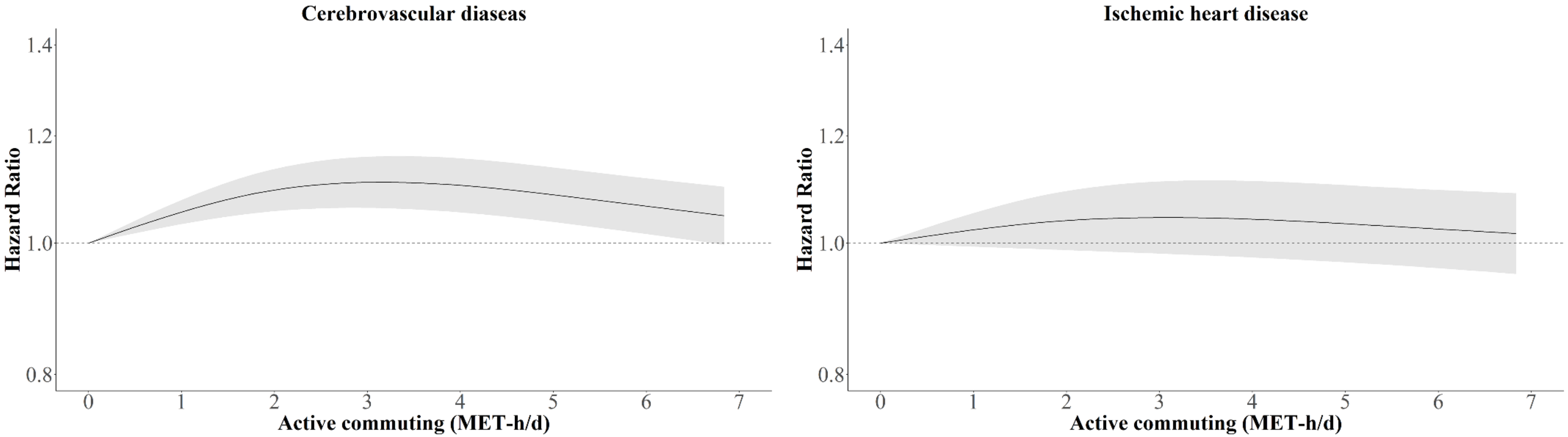

Figure S6. Associations of physical activity from active commuting with cerebrovascular disease and ischemic heart disease among farmers (n=204,125).

MET, metabolic equivalent of task. Solid lines represent hazard ratios and the ribbons represent 95% confidence intervals from Cox proportional hazard models with restricted cubic splines. Curves within the 95th percentile of active commuting level were shown.

The models were adjusted for sex, age (years), education, household income, seasonal work pattern of farm, smoking status, alcohol consumption, consumption of fresh vegetables, fresh fruits, and red meat (days per week), leisure sedentary time (hours per day), fuel types for cooking and heating in baseline house, cumulative exposure time to solid fuels in the past three houses (years), cookstove ventilation in the baseline house, duration of living with a smoker (years), exposure to secondhand smoke (hours/day), BMI (kg/m^2), WHR, self-rated health status, prevalent hypertension and diabetes, family histories of heart disease and stroke, and the remaining part after deducting the active commuting from the total physical activity (MET-h/d).

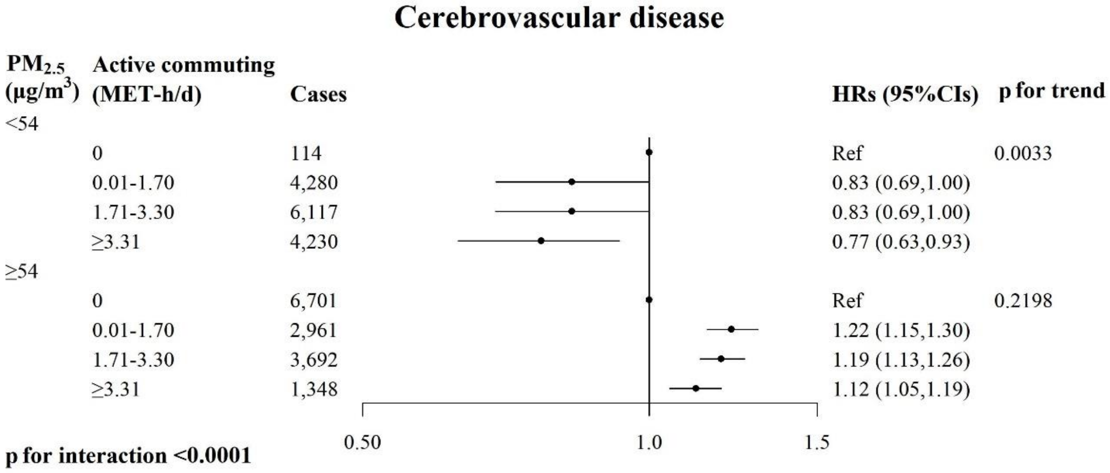

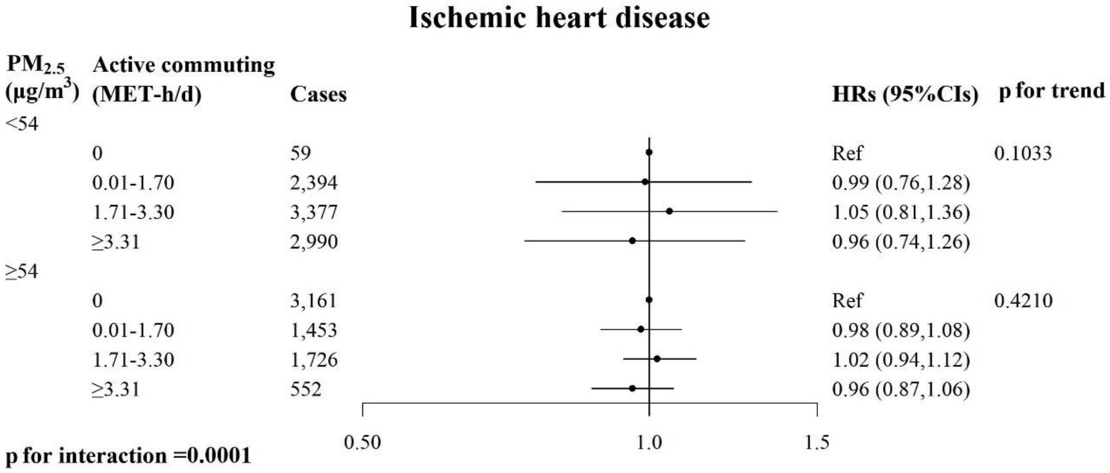

Figure S7. Associations of active commuting level categories with cerebrovascular disease and ischemic heart disease among farmers stratified according to the median of $PM_{2.5}$ concentration (n=204,125).

MET, metabolic equivalent of task.

The models were adjusted for sex, age (years), education, household income, seasonal work pattern of farm, smoking status, alcohol consumption, consumption of fresh vegetables, fresh fruits, and red meat (days per week), leisure sedentary time (hours per day), fuel types for cooking and heating in baseline house, cumulative exposure time to solid fuels in the past three houses (years), cookstove ventilation in the baseline house, duration of living with a smoker (years), exposure to secondhand smoke (hours/day), BMI (kg/m^2), WHR, self-rated health status, prevalent hypertension and diabetes, family histories of heart disease and stroke, and the remaining part after deducting the active commuting from the total physical activity (MET-h/d).

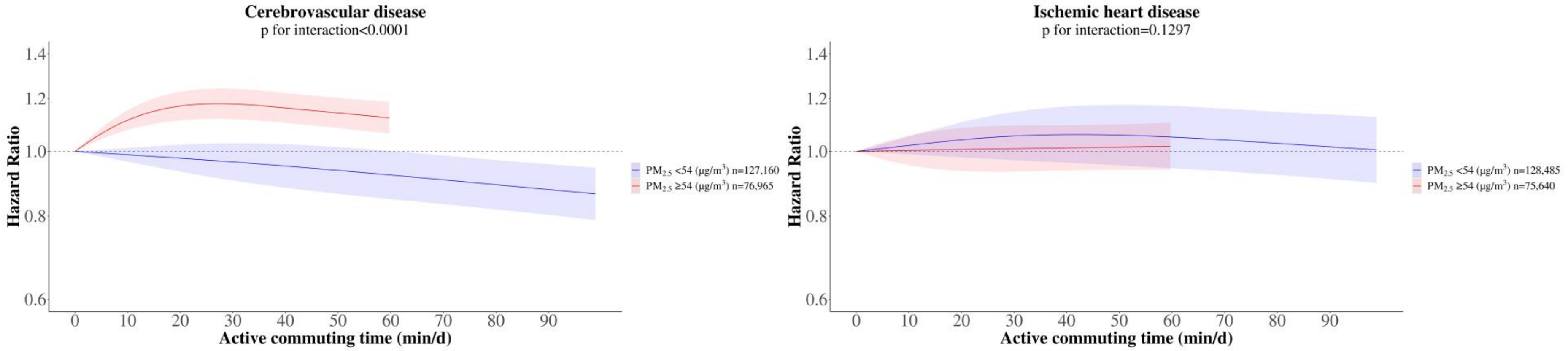

Figure S8. Associations of active commute time with cerebrovascular disease and ischemic heart disease among farmers stratified according to the median of $PM_{2.5}$ concentration (n=204,125).

MET, metabolic equivalent of task. Solid lines represent hazard ratios and the ribbons represent 95% confidence intervals from Cox proportional hazard models with restricted cubic splines. Curves within the 95th percentile of active commuting time in each stratum were shown.

The models were adjusted for sex, age (years), education, household income, seasonal work pattern of farm, smoking status, alcohol consumption, consumption of fresh vegetables, fresh fruits, and red meat (days per week), leisure sedentary time (hours per day), fuel types for cooking and heating in baseline house, cumulative exposure time to solid fuels in the past three houses (years), cookstove ventilation in the baseline house, duration of living with a smoker (years), exposure to secondhand smoke (hours/day), BMI (kg/m^2), WHR, self-rated health status, prevalent hypertension and diabetes, family histories of heart disease and stroke, and the remaining part after deducting the active commuting from the total physical activity (MET-h/d).

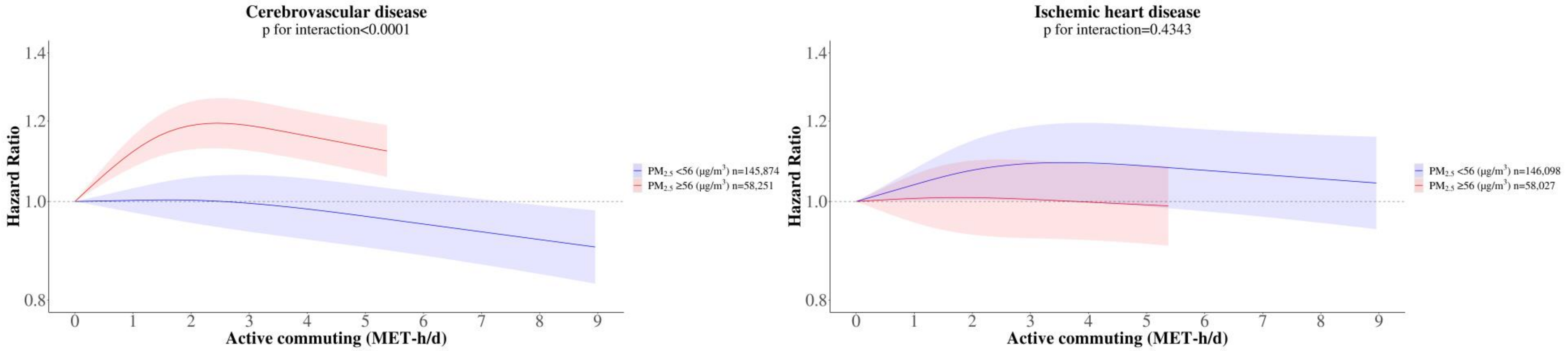

Figure S9. Associations of physical activity from active commuting with cerebrovascular disease and ischemic heart disease among farmers stratified according to the upper tertile of $PM_{2.5}$ concentration (n=204,125).

MET, metabolic equivalent of task. Solid lines represent hazard ratios and the ribbons represent 95% confidence intervals from Cox proportional hazard models with restricted cubic splines. Curves within the 95th percentile of active commuting level in each stratum were shown.

The models were adjusted for sex, age (years), education, household income, seasonal work pattern of farm, smoking status, alcohol consumption, consumption of fresh vegetables, fresh fruits, and red meat (days per week), leisure sedentary time (hours per day), fuel types for cooking and heating in baseline house, cumulative exposure time to solid fuels in the past three houses (years), cookstove ventilation in the baseline house, duration of living with a smoker (years), exposure to secondhand smoke (hours/day), BMI (kg/m^2), WHR, self-rated health status, prevalent hypertension and diabetes, family histories of heart disease and stroke, and the remaining part after deducting the active commuting from the total physical activity (MET-h/d).

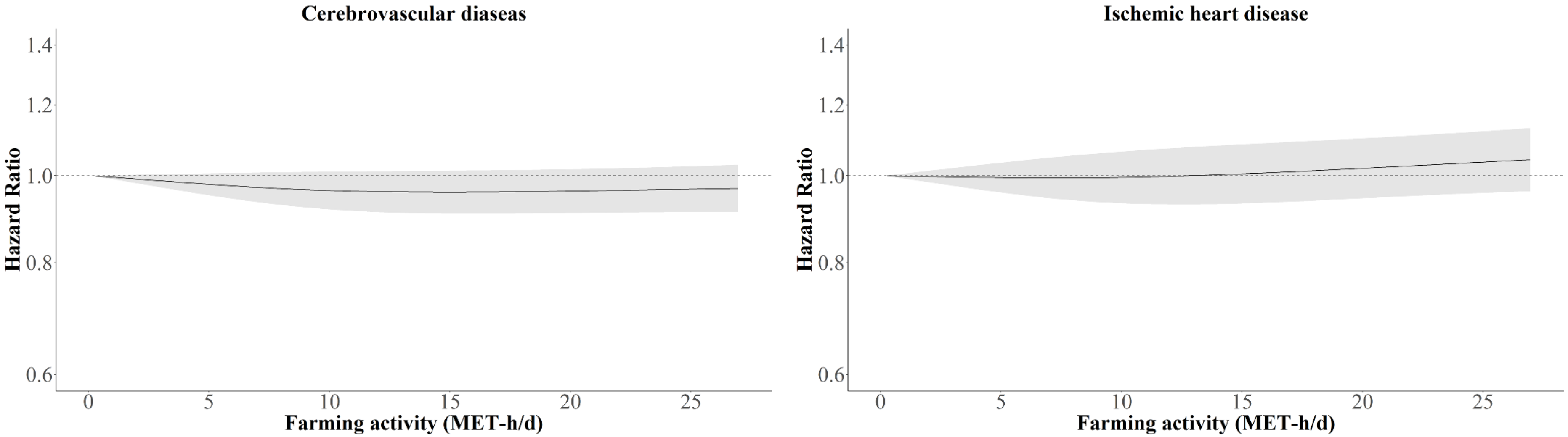

Figure S10. Associations of physical activity from farming with cerebrovascular disease and ischemic heart disease among farmers (n=201,140).

MET, metabolic equivalent of task. Solid lines represent hazard ratios and the ribbons represent 95% confidence intervals from Cox proportional hazard models with restricted cubic splines. Curves within the 95th percentile of farming activity level were shown.

The models were adjusted for sex, age (years), education, household income, seasonal work pattern of farm, smoking status, alcohol consumption, consumption of fresh vegetables, fresh fruits, and red meat (days per week), leisure sedentary time (hours per day), fuel types for cooking and heating in baseline house, cumulative exposure time to solid fuels in the past three houses (years), cookstove ventilation in the baseline house, duration of living with a smoker (years), exposure to secondhand smoke (hours/day), BMI (kg/m^2), WHR, self-rated health status, prevalent hypertension and diabetes, family histories of heart disease and stroke, and the remaining part after deducting the farming activity from the total physical activity (MET-h/d).

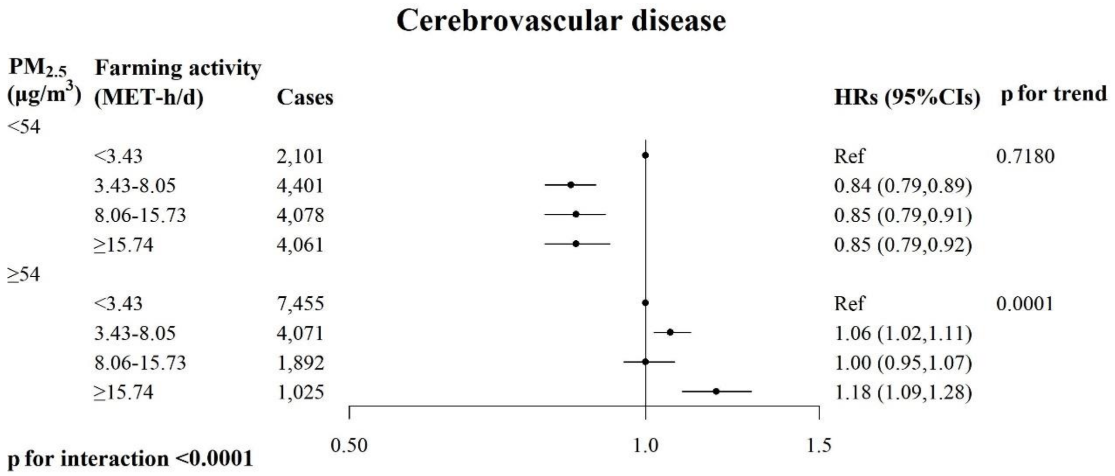

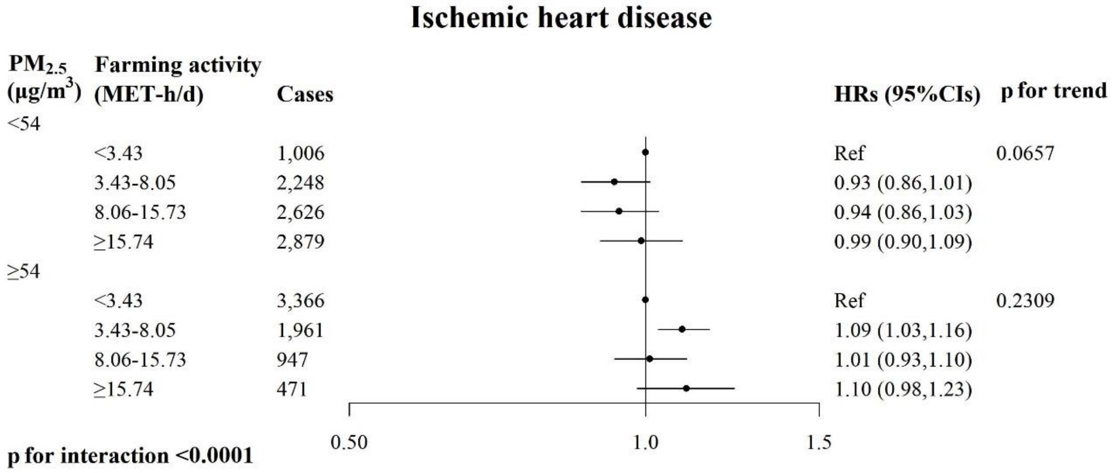

Figure S11. Associations of farming activity level categories with cerebrovascular disease and ischemic heart disease among farmers stratified according to the median of $PM_{2.5}$ concentration (n=201,140).

MET, metabolic equivalent of task.

The models were adjusted for sex, age (years), education, household income, seasonal work pattern of farm, smoking status, alcohol consumption, consumption of fresh vegetables, fresh fruits, and red meat (days per week), leisure sedentary time (hours per day), fuel types for cooking and heating in baseline house, cumulative exposure time to solid fuels in the past three houses (years), cookstove ventilation in the baseline house, duration of living with a smoker (years), exposure to secondhand smoke (hours/day), BMI (kg/m^2), WHR, self-rated health status, prevalent hypertension and diabetes, family histories of heart disease and stroke, and the remaining part after deducting the farming activity from the total physical activity (MET-h/d).

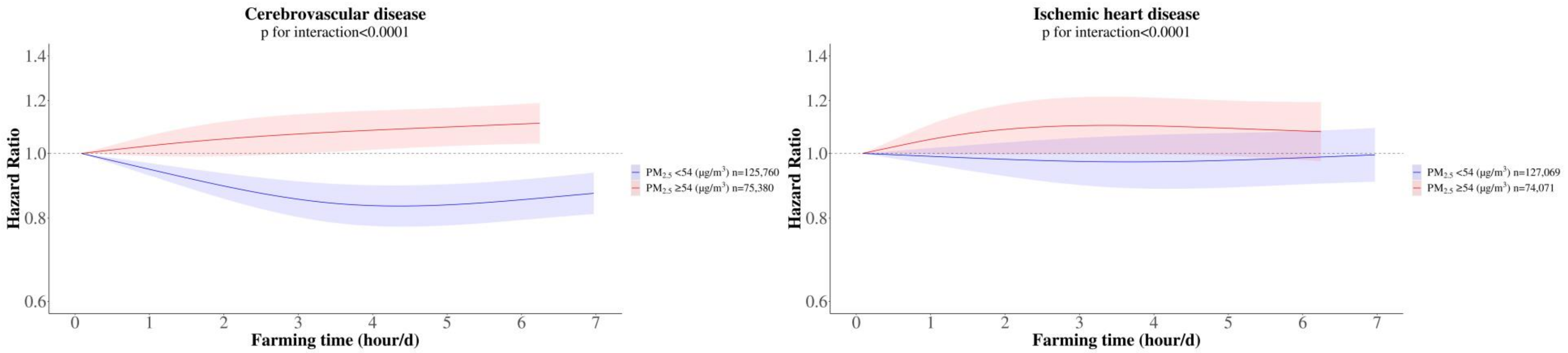

Figure S12. Associations of farming time with cerebrovascular disease and ischemic heart disease among farmers stratified according to the median of $PM_{2.5}$ concentration (n=201,140).

MET, metabolic equivalent of task. Solid lines represent hazard ratios and the ribbons represent 95% confidence intervals from Cox proportional hazard models with restricted cubic splines. Curves within the 95th percentile of farming time in each stratum were shown.

The models were adjusted for sex, age (years), education, household income, seasonal work pattern of farm, smoking status, alcohol consumption, consumption of fresh vegetables, fresh fruits, and red meat (days per week), leisure sedentary time (hours per day), fuel types for cooking and heating in baseline house, cumulative exposure time to solid fuels in the past three houses (years), cookstove ventilation in the baseline house, duration of living with a smoker (years), exposure to secondhand smoke (hours/day), BMI (kg/m^2), WHR, self-rated health status, prevalent hypertension and diabetes, family histories of heart disease and stroke, and the remaining part after deducting the farming activity from the total physical activity (MET-h/d).

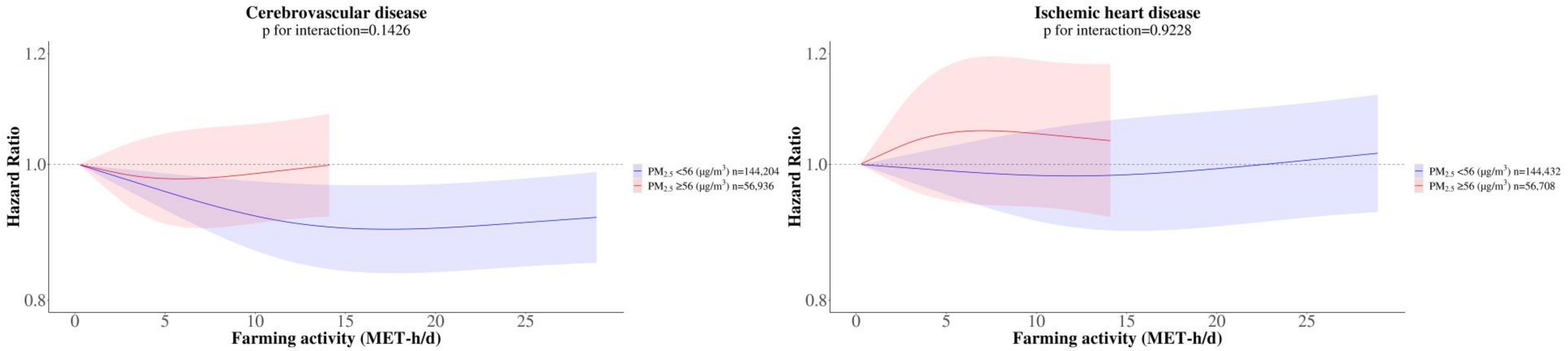

Figure S13. Associations of physical activity from farming with cerebrovascular disease and ischemic heart disease among farmers stratified according to the upper tertile of $PM_{2.5}$ concentration (n=201,140).

MET, metabolic equivalent of task. Solid lines represent hazard ratios and the ribbons represent 95% confidence intervals from Cox proportional hazard models with restricted cubic splines. Curves within the 95th percentile of farming activity level in each stratum were shown.

The models were adjusted for sex, age (years), education, household income, seasonal work pattern of farm, smoking status, alcohol consumption, consumption of fresh vegetables, fresh fruits, and red meat (days per week), leisure sedentary time (hours per day), fuel types for cooking and heating in baseline house, cumulative exposure time to solid fuels in the past three houses (years), cookstove ventilation in the baseline house, duration of living with a smoker (years), exposure to secondhand smoke (hours/day), BMI (kg/m^2), WHR, self-rated health status, prevalent hypertension and diabetes, family histories of heart disease and stroke, and the remaining part after deducting the farming activity from the total physical activity (MET-h/d).
